# Pediatric Endoscopic Pilonidal Sinus Treatment (PEPSiT) in Children With Pilonidal Sinus Disease: Tips and Tricks and New Structurated Protocol

**DOI:** 10.3389/fped.2020.00345

**Published:** 2020-06-24

**Authors:** Ciro Esposito, Mario Mendoza-Sagaon, Fulvia Del Conte, Mariapina Cerulo, Vincenzo Coppola, Giovanni Esposito, Giuseppe Cortese, Felice Crocetto, Ernesto Montaruli, Maria Escolino

**Affiliations:** ^1^Pediatric Surgery Unit, Federico II University of Naples, Naples, Italy; ^2^Pediatric Surgery Unit, Ospedale Regionale Bellinzona e Valli, Bellinzona, Switzerland

**Keywords:** pilonidal sinus disease, children, PEPSiT, laser, dressing

## Abstract

**Background:** The advent of pediatric endoscopic pilonidal sinus treatment (PEPSiT) has dramatically changed the surgical management of pilonidal sinus disease (PSD) in children and adolescents. This study aimed to report the outcome of our new structurated protocol, including PEPSiT, laser epilation, and oxygen-enriched oil-based gel dressing, for treatment of PSD in pediatric patients and describe tips and tricks of the technique.

**Methods:** We retrospectively reviewed the data of 127 pediatric patients, who underwent PEPSiT for PSD in our institutions over a 36-month period. All patients received laser epilation (LE) before and after surgery. Post-operative dressing was performed using silver sulfadiazine spray and in the last 18 months oxygen-enriched oil-based gel. We divided the patients in two groups according to the protocol adopted: G1 (laser + oxygen-enriched oil-based gel dressing) included 72 patients and G2 (laser + silver sulfadiazine spray dressing) included 55 patients. The two groups were compared regarding success rate, recurrence, wound infection rate, wound healing time, post-operative outcome, time to full daily activities and patient satisfaction.

**Results:** No difference emerged between the two groups regarding the average operative time, the average post-operative pain score, the average analgesic requirement, the average hospitalization and the average time to full daily activities (*p* = 0.33). No intra- or post-operative complications including wound infection occurred in both groups. The patients required an average number of 7 LE sessions (range 4–10) to achieve complete hair removal. The overall success rate was significantly higher in G1 (*n* = 71, 98.6%) compared with G2 (*n* = 50, 90.9%) [*p* = 0.001]. The recurrence rate was also significantly lower in G1 (*n* = 1, 1.4%) compared with G2 (*n* = 5, 9%) [*p* = 0.001]. Furthermore, G1 reported a faster wound healing (average 21 days) compared with G2 (average 29 days) [*p* = 0.001] and a higher patient satisfaction score (average 4.9) compared with G2 (average 4.2) [*p* = 0.001].

**Conclusions:** Based upon our experience, PEPSiT may be considered the standard of care for surgical treatment of PSD in children and adolescents. Our new structurated protocol consisting of pre-operative LE, PEPSiT, and post-operative wound management with oxygen-enriched oil-based gel dressing and LE, allowed to achieve an excellent outcome, with a success rate > 98%.

## Introduction

Pilonidal sinus disease (PSD) is a common disabling inflammatory disease, that mainly affects teenagers and young adults with higher incidence in male gender (1, 2). An important predisposing factor is the presence of hair in the intergluteal crease (3). Other known risk factors include familiarity, repeated local traumatisms, sedentary life, and obesity (4). PSD is now considered an acquired disease, that is mainly due to a chronic inflammatory response to the retention of hair follicles into the intergluteal cleft causing formation of abscesses and usually multiple fistula's tracts (5). The disease may have a very negative impact on quality of life of affected subjects, causing long absences from work or school activities.

The treatment of this pathology remains controversial; several techniques, including excision and packing, excision and primary closure, marsupialization, and flap procedures, have been described with different success rates but the gold standard approach has not yet been established (6, 7). The main problem with surgical treatment, independently from the technique, was the high recurrence rate, up to 30% in some series (1). This high recurrence rate has been mainly attributed to the persistence of hair near the surgical site (1). Furthermore, traditional surgery was associated with a very long and painful post-operative course. So, minimally invasive alternatives to open surgery, including radiosurgery, fibrin glue injection and more recently endoscopy, have been recently adopted for treatment of PSD (8–11).

We modified the endoscopic technique, first described by Meinero in adults (10), in order to apply it in the pediatric population and we called it PEPSiT (Pediatric Endoscopic Pilonidal Sinus Treatment) (12). After a 3-year experience with PEPSiT, we believe that the key factor to achieve the complete healing of PSD was not only the standardization of the surgical procedure but also pre- and post-operative laser epilation and accurate post-operative wound treatment (13).

In a previous paper, we described our protocol for wound dressing that included application of 2% eosin solution and silver sulfadiazine spray (13). In the last 18 months we introduced use of a new product, an oxygen-enriched oil-based gel, instead of silver sulfadiazine spray, for post-operative wound dressing.

This study aimed to report the outcome of our new structurated protocol, including PEPSiT, laser epilation and oxygen-enriched oil-based gel dressing, for treatment of PSD in pediatric patients, to compare it with our previous protocol and finally to describe tips and tricks of the technique.

## Materials and Methods

We retrospectively reviewed the data of 127 consecutive pediatric patients, 55 girls and 72 boys, who underwent PEPSiT for PSD in our institutions over a 36-month period. All the patients presented with acute or chronic pilonidal fistula and 15 out of 127 patients (11.8%) had a recurrent PSD following open repair performed in another center. Additionally, 7/127 (5.5%) patients presented a concomitant pilonidal cyst.

All PEPSiT procedures were performed by a single pediatric surgeon, who mastered proficiently the technique. Follow-up included outpatient evaluation at 1, 2, and 4 weeks post-operatively, then every 3 months until 18 months after surgery and thereafter once a year.

All patients received laser epilation before and after surgery. Post-operative dressing was performed using silver sulfadiazine spray and in the last 18 months oxygen-enriched oil-based gel. We divided the patients in two groups according to the protocol adopted: G1 (laser + oxygen-enriched oil-based gel dressing) included 72 patients and G2 (laser + silver sulfadiazine spray dressing) included 55 patients. The two groups were compared regarding success rate, recurrence of disease, wound infection rate, wound healing time, post-operative outcome, time to full daily activities and patient satisfaction.

The surgical success rate was defined as the complete wound healing during the first 60 post-operative days. Recurrence of disease was considered when symptoms and/or secretion occurred after any interval following wound healing. Post-operative pain was scored using the visual analog scale (VAS). Patients were asked to score their satisfaction about post-operative course of PEPSiT using a 5-items Likert-type scale (1–5), with 1 = very poor; 2 = poor; 3 = average; 4 = good; 5 = excellent.

Statistical analysis was carried out by using the Statistical Package for Social Sciences (SPSS Inc., Chicago, Illinois, USA), version 13.0. Demographic data were compared using the Student's *t-*test. The categorical variables were compared using χ^2^ tests. Significance was defined as *p* < 0.05.

The study received appropriate Institute Review Board (IRB) approval at Federico II University in Naples, Italy. All subjects gave written informed consent in accordance with the Declaration of Helsinki.

In the last 18 months we standardized a new PEPSiT protocol that included 3 steps, described in detail as follows.

### Step 1—Pre-operative Management

**Figure 1:**
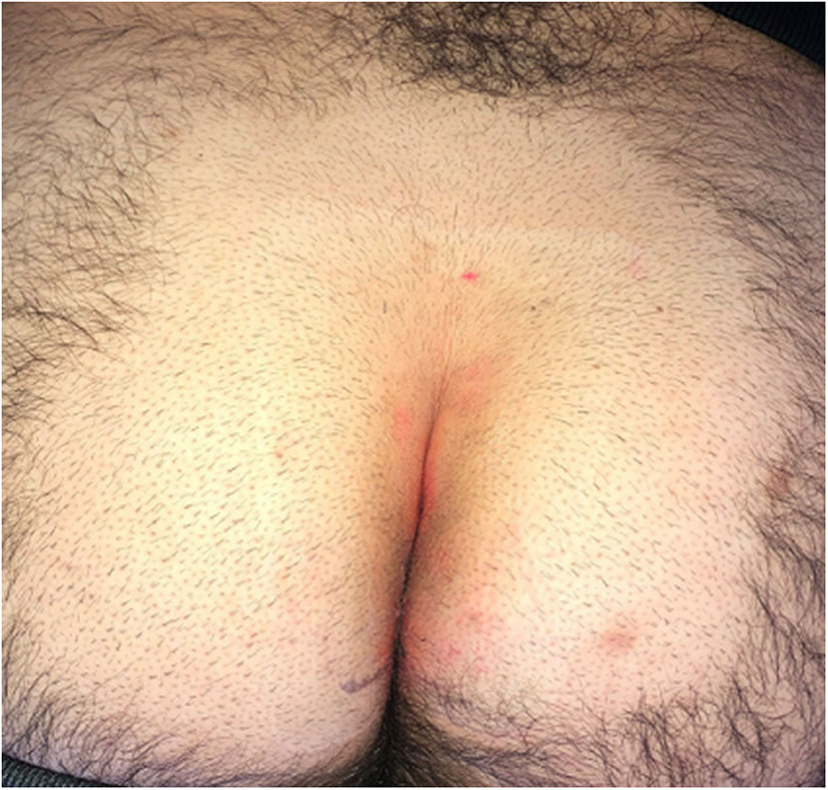
Laser epilation therapy included the natal cleft and an additional 5 cm area on both sides (left/right).

**Figure 2:**
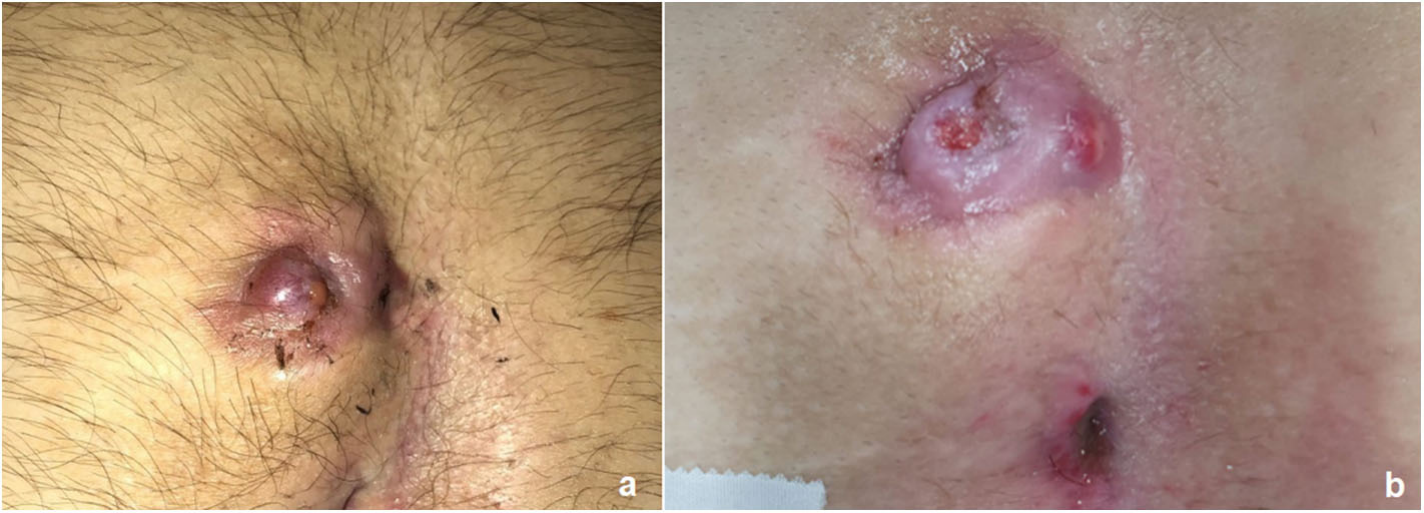
Outcome of pre-operative laser epilation: before **(a)** and after 2 sessions **(b)**.

All patients underwent pre-operatively a laser epilation (LE) treatment of the intergluteal region. A pulse-dye laser was adopted in all patients, with a setting of 4.0 pulse on and 20–25 J/cm^2^. LE treatment was performed in the affected area of the intergluteal crease including an additional 5 cm area on both sides (left/right) of the natal cleft (Figure 1). Each patient underwent at least 2–3 pre-operative LE sessions at 4–6 week intervals, according to their local status (Figure 2). Each LE session lasted average 10 min and was well-tolerated by all patients.

### Step 2—Surgical Treatment

All patients and their parents signed a specific informed consent before surgery. All surgical procedures were performed using a fistuloscope, a monopolar electrode, an endobrush and an endoscopic grasping forceps. The 10 Ch fistuloscope, manufactured by Karl Storz, has an 8° angled eyepiece and is equipped with an optical channel and a working and irrigation channel. Its diameter is 3.2 × 4.8 mm, and its operative length is 18 cm. A removable handle allows easier maneuvering and better ergonomics for the surgeon, the handle can be moved in different positions according to the surgeon's preference.

Patients received a specific type of subarachnoid anesthesia and antibiotic prophylaxis. The patients were placed in prone position with buttocks retracted using adhesive tape. Surgeon's position was at the patient's right. Two screens were placed, one at patient's feet and one at patient's head, to be adopted alternatively according to the lower or upper extension of the fistula. The 5-step operative technique, that we already described (12, 13), was adopted in all cases. In the 1st step, the fistuloscope was introduced through the fistula's external opening. If there were multiple pits, the lower one was used to introduce the fistuloscope. If the orifice was too small, it was enlarged using a spreading clamp or urethral dilators and the entire tract was enlarged by injecting saline with a syringe. In the 2nd step, we identified the anatomy of pilonidal sinus and any lateral tracts and/or abscess cavities thanks to a continuous jet of irrigation solution. In the 3rd step, all the hairs and bulbs were removed under vision using endoscopic grasping forceps. In the 4th step, the sinus tract was debrided using the endobrush and any residual granulation tissue was eliminated by the solution flow.

Finally, during the 5th step, a cautery ablation of the sinus granulation tissue was performed using the monopolar electrode, commencing in the main tract and where appropriate traversing secondary tracts and abscess cavities. Particular attention was paid to hemostasis during the procedure. External opening(s) were not closed and were covered by a compression dressing. In the last 10 patients, the external openings were cauterized using laser energy in order to try to fasten the healing of the fistula's orifices (Pediatric Endoscopic Pilonidal Sinus Laser Treatment—PEPSiLaT). No drain was placed at the end of the procedure.

All operative steps are reported in Video 1.

In 7 patients, we found, beside the fistula, a concomitant pilonidal cyst, that was always located laterally to the fistula's opening. The cysts were removed performing a small incision and then sent for histological examination.

### Step 3—Post-operative Management

Oral intake was allowed 1 h after operation and early ambulation 2–3 h after surgery was encouraged. All patients could keep a normal decubitus in the immediate post-operative period. Following hospital discharge, the patients were instructed to treat the wound daily by applying a 2% eosin topical solution and an oxygen-enriched oil-based gel, that was directly injected within the fistula's tract. Thereafter, the wound was covered with a wet gauze and a normal dressing was finally performed. These dressings were repeated at least 2 times/day for at least 2–3 weeks post-operatively. The specific dressing that we performed in patients who underwent PEPSiT is shown in Video 2.

**Figure 3:**
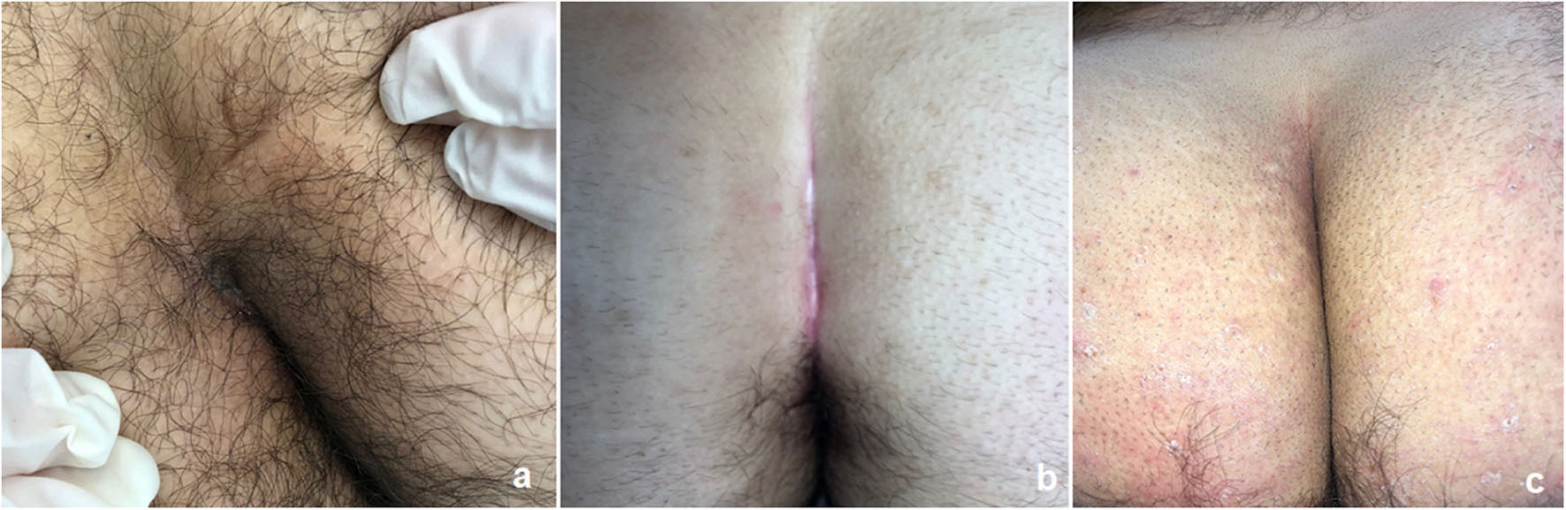
Outcome of post-operative laser epilation: before **(a)** and after 3 sessions **(b)** and 6 sessions **(c)**.

As soon as the wound healing was complete, all patients underwent radical LE treatment of the intergluteal region using pulse-dye laser. LE treatment was performed in the affected area of the intergluteal crease including an additional 5 cm area on both sides (left/right) of the natal cleft. Each patient received at least 5–7 post-operative LE sessions at 4–6 week intervals, according to their local status. Completion of the LE treatment was defined as no visible hair in the treatment area (Figure 3).

## Results

The two groups were statistically homogeneous regarding the male/female ratio (G1 = 40/32 vs. G2 = 32/23, *p* = 0.33), the average patients' age (G1 = 16.3 years vs. G2 = 15.8 years, *p* = 0.55) and the average patients' weight (G1 = 75.5 Kgs vs. G2 = 71.5 Kgs, *p* = 0.33).

All patients' demographics in G1 and G2 are reported in Table 1.

**Table 1:** Patients' demographics in G1 and G2.

**Patients' demographics**	**G1 (laser + oxygen-enriched oil-based gel)**	**G2 (laser + silver sulfadiazine spray)**	***P*-value**
Number of patients, n	72	55	0.33
Male/female, n/n	40/32	32/23	0.55
Average age, years (range)	16.3 (14–18)	15.8 (13–18)	0.55
Average weight, Kgs (range)	75.5 (58–102)	71.5 (55–100)	0.33
Recurrent PSD, n (%)	9 (12.5%)	6 (10.9%)	0.55
Pilonidal cyst, n (%)	4 (5.5%)	3 (5.4%)	0.55

*PSD, pilonidal sinus disease*.

No difference emerged between the two groups regarding the average operative time (G1 = 23 min vs. G2 = 27 min, *p* = 0.33), the average post-operative VAS pain score, assessed at 24 h post-operatively (G1 = 1.7 vs. G2 = 1.8, *p* = 0.33), the average analgesic requirement (G1 = 14 h vs. G2 = 16 h, *p* = 0.33), the average hospitalization (G1 = 20 h vs. G2=19 h, *p* = 0.33) and the average time to full daily activities (G1 = 2 days vs. G2 = 2 days, *p* = 0.33).

No intra- or post-operative complications including wound infection occurred in both groups. No patients had any physical limitations post-operatively. All patients of both groups required an average number of 7 laser epilation sessions (range 4–10) to achieve complete hair removal. No patients had any apparent complications related to the LE, such as skin pigmentation changes or thermal injuries.

**Figure 4:**
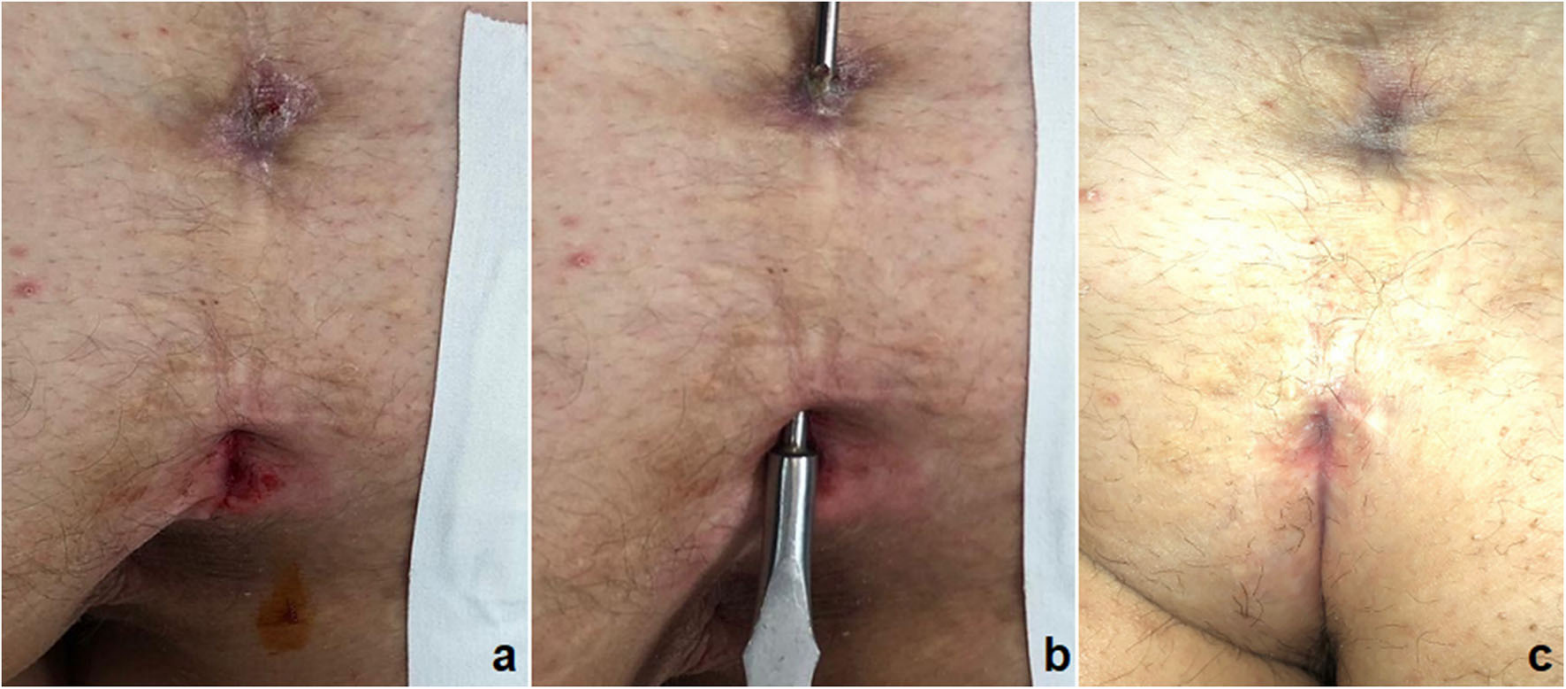
Outcome of PEPSiT in a recurrent PSD: before **(a,b)** and 28 days post-operatively **(c)**.

**Figure 5:**
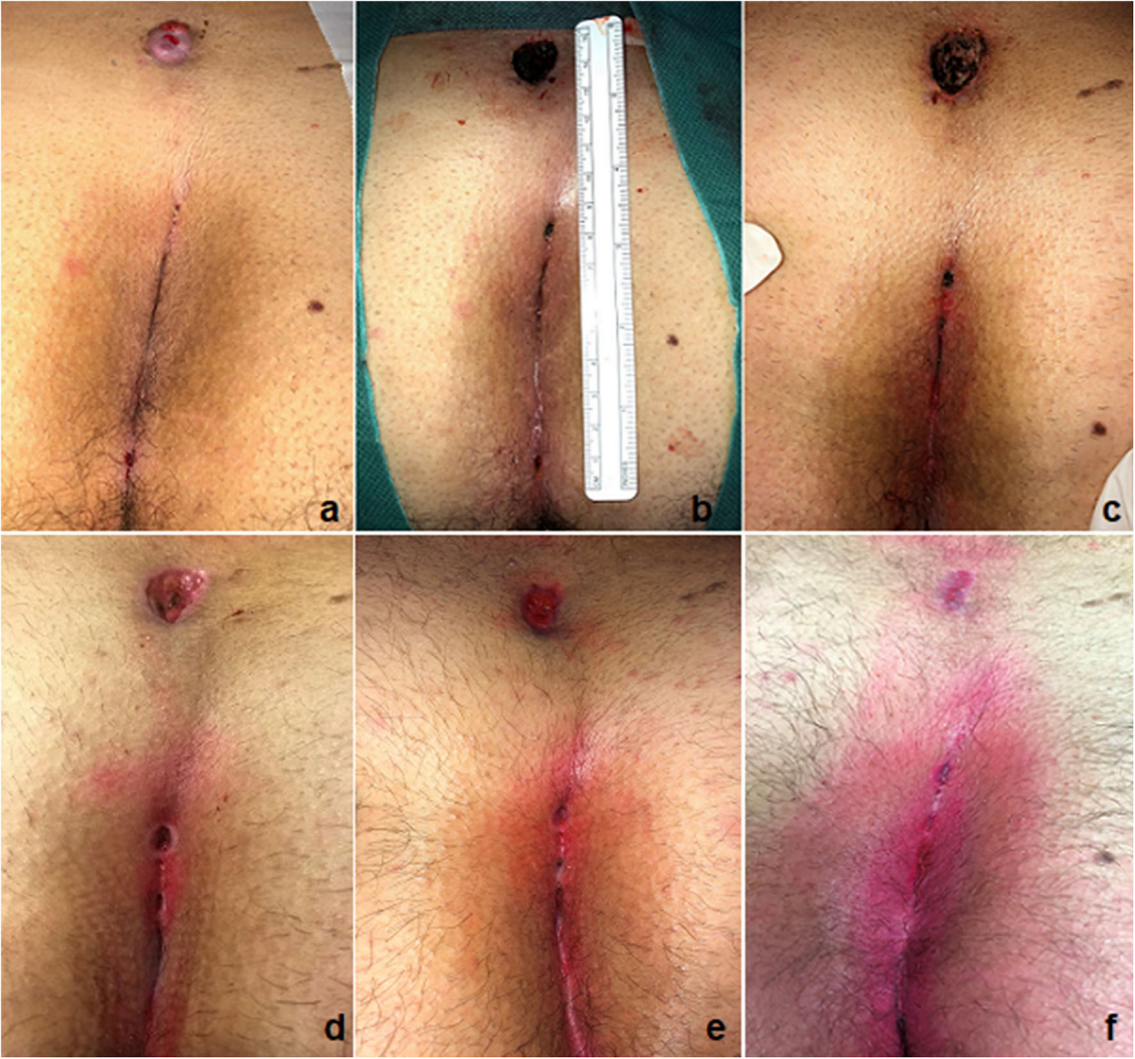
Outcome of PEPSiT in a 15-cm long PSD: before **(a)**, intra-operatively **(b)**, 7 days **(c)**, 15 days **(d)**, 25 days **(e)**, and 32 days **(f)** post-operatively.

The overall success rate was significantly higher in G1 (*n* = 71, 98.6%) compared with G2 (*n* = 50, 90.9%) [*p* = 0.001]. The recurrence rate was also significantly lower in G1 (*n* = 1, 1.4%) compared with G2 (*n* = 5, 9%) [*p* = 0.001]. All recurrent PSD were re-operated using PEPSiT, with no further recurrence. Furthermore, G1 reported a faster wound healing (average 21 days) compared with G2 (average 29 days) [*p* = 0.001] and a higher patient satisfaction score (average 4.9) compared with G2 (average 4.2) [*p* = 0.001]. All patients of our series regarded this technique favorably due to the painless post-operative course and the excellent cosmetic results (Figures 4, 5). Moreover, G1 patients reported higher satisfaction scores compared with G2 patients due to the quicker wound healing and subsequent shorter needing for post-operative wound dressings.

Comparative outcome between G1 and G2 is reported in Table 2.

**Table 2:** Comparative outcome between G1 and G2 in our series.

**Parameter**	**G1 (*n =* 72) (laser + oxygen-enriched oil-based gel)**	**G2 (*n =* 55) (laser + silver sulfadiazine spray)**	***P*-value**
Average operative time, minutes (range)	23 (18–50)	27 (20–67)	0.33
Intra-operative complications, n	0	0	n/a
Average VAS score at 24 h post-operatively, n (range)	1.7 (1–3)	1.8 (1–4)	0.33
Average analgesic requirement, hours (range)	14 (6–20)	16 (8–22)	0.33
Average hospital stay length, hours (range)	20 (16–36)	19 (12–34)	0.33
Average time to full daily activities, days (range)	2 (1–4)	2 (1–4)	0.33
Average number of LE sessions, n (range)	7 (4–10)	7 (4–10)	0.33
Wound infection, n (%)	0	0	n/a
Recurrence, n (%)	1 (1.4%)	5 (9%)	0.001
Overall success rate, n (%)	71 (98.6%)	50 (90.9%)	0.001
Average healing time, days (range)	21 (17–28)	29 (22–40)	0.001
Average patient satisfaction score (1–5), n (range)	4.9 (3.9–5)	4.2 (4–5)	0.001

*n/a, not applicable; LE, laser epilation*.

## Discussion

We started to adopt PEPSiT in pediatric patients in 2016 (12); after this date, the number of patients requiring this technique has increased exponentially. In pre-PEPSiT era we usually operated 3–4 patients with PSD per year using open approach. After the advent of endoscopic procedure, PEPSiT, we currently operate 30–40 patients per year. We believe that the incidence of PSD is probably underestimated and before the advent of PEPSiT patients preferred to live with untreated pathology in order to avoid the high morbidity and the long post-operative course of open repair.

We modified the endoscopic technique, first described by Meinero in adults, to adapt it to pediatric patients and we renamed it PEPSiT (Pediatric Endoscopic Pilonidal Sinus Treatment) (12). We already published our preliminary experience with the technique in two single-center studies and in an Italian multicentric survey (12–14). After a 36-month experience, we have standardized the steps of the technique (13) and we obtained a success rate higher than 95% but with a painless post-operative course and a very short hospital stay.

Based upon our experience, the key factors for the success of the technique are the use of PEPSiT with the advantages and the precision of endoscopic surgery but also the laser epilation treatment before and after surgery and the post-operative wound management that allowed us to report a very low recurrence rate (<5%). As we already published (13), we believe that it is crucial to standardize not only the procedure itself, but also the pre-operative preparation and the post-operative management.

Considering that hirsutism is one of the main risk factors of the disease and the persistence of hair near the surgical site is considered the main cause of post-operative recurrence (1, 3), strategies of hair removal in the natal cleft have been applied in efforts to reduce recurrence of PSD (15, 16). Different methods such as electrolysis, depilatory creams and shaving the area have been used for hair removal reporting short-term success, poor compliance and high recurrence rate (17). Laser technology represents the best way to obtain a radical hair removal in the treatment area (18–20). A recent systematic review of the literature showed a reduced recurrence rate after laser hair removal (9.3%) performed after surgical treatment of PSD, compared to no hair removal (19.7%) and razor and cream depilation (23.4%) (21). In our series, all patients received a laser epilation treatment of the pilonidal region that started before surgery and was continued post-operatively, after complete wound healing, every month until there was no longer any visible hair in the treatment area.

**Figure 6:**
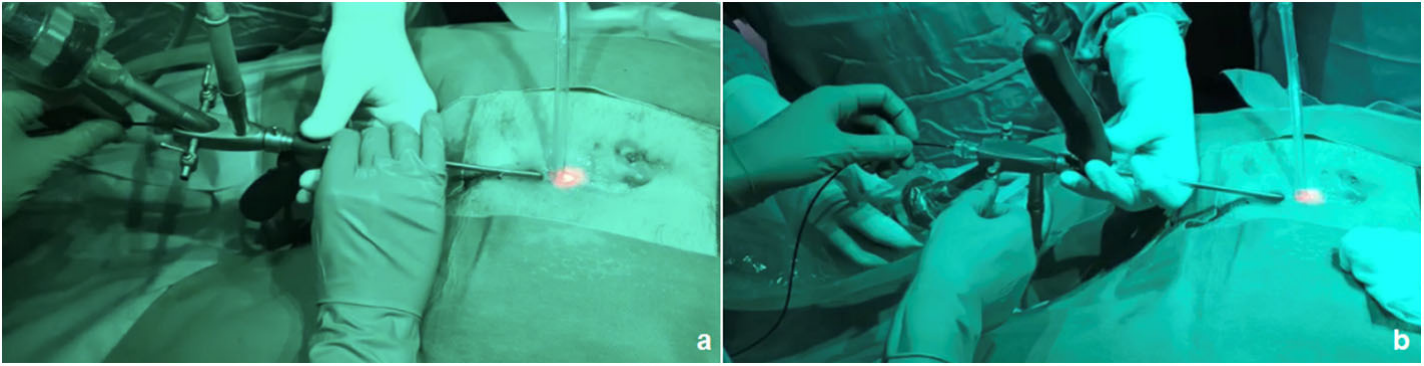
The fistuloscope may be used in normal position **(a)** or turned 180 degrees down **(b)** in order to achieve a good view of the fistula's roof.

Regarding the operative technique, we already standardized PEPSiT as a 5-step procedure (12, 13) and we can outline some tips and tricks based upon our experience. A key point is the use of the fistuloscope, that can be easily managed using the handle and rotated in different angles in order to improve the intra-operative visualization and ease the procedure. Another trick we used was to turn the fistuloscope (that has a 30° down angulation) 180 degrees down in order to have a perfect view of the fistula's roof (Figure 6). Using this trick, we were able to visualize some hairs located on the roof, that would be missed using the normal position of the fistuloscope. Before introducing the fistuloscope through the fistula's tract, the external opening if too tight was enlarged using a spreading clamp or urethral dilators in order to allow the introduction of the 10 Ch fistuloscope, then saline solution was injected under pression with a syringe through the external pit in order to enlarge and wash the cavity. During surgery we adopted both normal saline or mannitol/sorbitol 0.54%/2.7% as irrigation solutions. In our experience, both methods gave a similar good intra-operative view and allowed an effective use of monopolar coagulation, although it has been reported in adults that use of mannitol solution increases the efficacy of monopolar coagulation (10). In presence of a concomitant pilonidal cyst, that was always located laterally to the fistula's external opening, an additional small incision should be performed to remove it. In the last few cases we adopted Laser energy (PEPSiLaT) to coagulate the fistula's external orifice(s) in order to fasten the healing process. We reported excellent results with this technique but these preliminary data need to be confirmed by larger series and longer follow-up.

In our experience, PEPSiT procedure demonstrated to have many advantages compared with traditional open techniques (12). First, the direct vision allowed the surgeon to see perfectly not only the pilonidal sinus, but also any possible fistula tracts or abscess cavities. Moreover, the hemostasis, that is one of the main problems of traditional open treatment, was done perfectly thoroughly under direct vision. This direct vision also allowed the complete removal of all hairs and their follicles, often located not only in the pilonidal sinus, but also in the surrounding tissue.

Another crucial point was the post-operative wound management. We believe that the successful outcome of PEPSiT in our series was not only related to the operative technique itself but especially to careful attention paid to wound management and local hygiene post-operatively. The rationale of dressing is to keep the wound clean and create the conditions to improve the healing process. In a previous paper, we described our protocol for wound dressing that included application of 2% eosin solution and silver sulfadiazine spray (13). In the last 18 months we introduced use of a new product, an oxygen-enriched oil-based gel, instead of silver sulfadiazine spray.

Ozone (O_3_) has been widely recognized as one of the best bactericidal, antiviral and antifungal agents and has been used empirically for post-surgical fistulas and acute wounds as well as chronic wounds such as trophic ulcers, ischemic ulcers, diabetic wounds, psoriasis (22, 23). The O_3_ is employed as ozonated oil, which is ideal for topical use in mucosal and cutaneous areas of the body. The ozonated compositions have the capacity to deliver nascent oxygen deep within the lesion without causing skin irritation (23). Ozonated gel oil has a double action on wound healing: decreased bacterial infection and increased oxygen tension by O_3_ exposure in the wound area (24). The beneficial effects of ozone on wound healing also include debridement effect, modulation of the inflammatory phase, stimulation of angiogenesis as well as biological and enzymatic reactions that favor oxygen metabolism improving wound healing (24, 25). Ozone activates the antioxidant system and has been reported as an effective therapeutic agent in the treatment of diabetes and its complication, demonstrating improved glycemic control, decreased oxidative stress, normalized organic peroxide levels and activated superoxide dismutase (26).

Based upon this available evidence, we decided to adopt oxygen-enriched oil-based gel in our patients who underwent PEPSiT and our study is the first report in the pediatric literature demonstrating the safe and effective use of oxygen-enriched oil-based gel for wound healing in pilonidal disease. However, it is not the first application in the pediatric population. We recently published a paper focused on endoscopic treatment of hidradenitis suppurativa, reporting use of oxygen-enriched oil-based gel for post-operative wound dressing (27). In such indications, the product was very effective allowing quick wound healing and clinically safe, with no adverse reactions or intolerance to the product.

Our results showed that use of oxygen-enriched oil-based gel in PSD was associated with a significantly faster wound healing and lower recurrence rate compared with our previous protocol based on silver sulfadiazine spray dressing. Furthermore, the decreased wound healing time allowed by oxygen-enriched oil-based gel was associated with higher satisfaction of patients, who had to perform dressings at home for a less time compared with the previous protocol. Based upon our experience, we strongly recommend inject the oxygen-enriched oil-based gel directly within the fistula's tract using a syringe in order to obtain a higher concentration of the product in the wound area and fasten the healing process.

Obviously, these preliminary results need to be confirmed by longer follow-up and larger series number.

**Figure 7:**
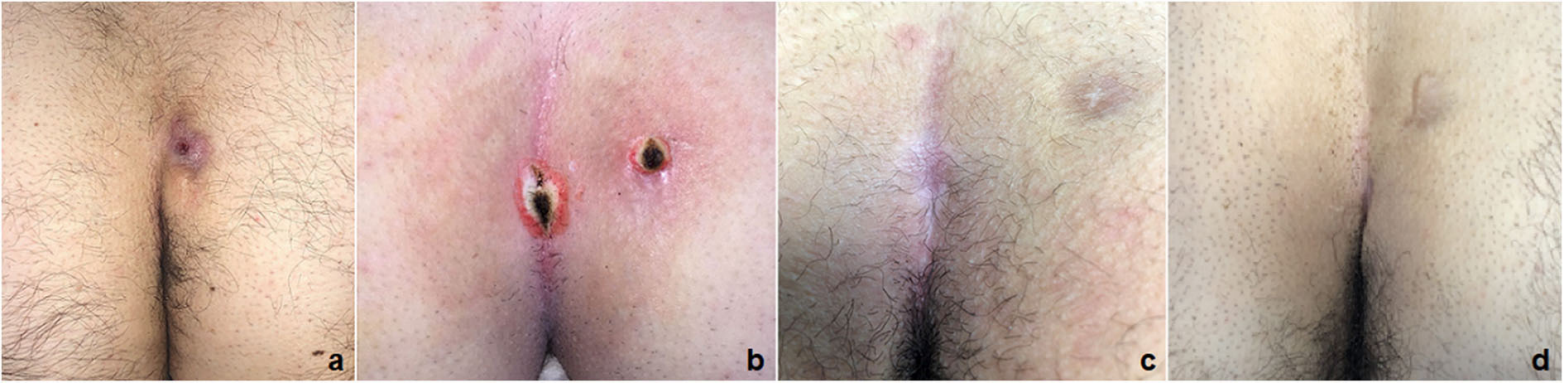
PEPSiT protocol steps: 1. Pre-operative laser epilation **(a)**; 2. PEPSiT **(b)**; 3. Post-operative laser epilation **(c)**; Final result **(d)**.

The standardization of new PEPSiT protocol allowed to obtain a success rate higher than 98%, with only 1.4% recurrence that was re-treated using the same technique with no further recurrence (Figure 7). The aesthetic result was excellent and so as the patient's quality of life and satisfaction (28).

Based upon our experience, we believe that PEPSiT may be considered the standard of care for surgical treatment of PSD in children and adolescents. Our new structurated protocol consisting of pre-operative laser epilation, PEPSiT procedure and post-operative wound management with oxygen-enriched oil-based gel dressing and laser epilation, allowed to achieve an excellent outcome, with a success rate > 98%. According with the patients' satisfaction, we believe that the open technique should be definitively abandoned for its hyper invasiveness and the very bad post-operative course.

## Data Availability Statement

All datasets generated for this study are included in the article/Supplementary Material.

## Author Contributions

CE contributed conception and design of the study and wrote the first draft of the manuscript. MM-S contributed conception and design of the study and wrote sections of the manuscript. FD, MC, VC, GE, GC, FC, EM, and ME organized the database and wrote sections of the manuscript. All authors contributed to the article and approved the submitted version.

## Conflict of Interest

The authors declare that the research was conducted in the absence of any commercial or financial relationships that could be construed as a potential conflict of interest.
